# The Body Mass Index and Waist Circumference as Predictors Of Body Composition in Post CSCI Wheelchair Rugby Players (Preliminary Investigations)

**DOI:** 10.2478/hukin-2014-0105

**Published:** 2014-11-12

**Authors:** Anna Zwierzchowska, Marta Głowacz, Agnieszka Batko-Szwaczka, Joanna Dudzińska-Griszek, Aleksandra Mostowik, Miłosz Drozd, Jan Szewieczek

**Affiliations:** 1 Department of Special Physical Education, Academy of Physical Education, Katowice.; 2 Department of Geriatrics, Medical University of Silesia.; 3 Department of Sports Theory, Academy of Physical Education, Katowice.

**Keywords:** spinal cord injury, obesity, visceral fat, wheelchair rugby players

## Abstract

The enforced sedentary lifestyle and muscle paresis below the level of injury are associated with adipose tissue accumulation in the trunk. The value of anthropometric indicators of obesity in patients with spinal cord injuries has also been called into question. We hypothesized that the Body Mass Index recommended by the WHO to diagnose obesity in general population has too low sensitivity in case of wheelchair rugby players.

The study group comprised 14 wheelchair rugby players, aged 32.6 ± 5.1 years, who had sustained CSCI (paralysis of lower limbs and upper extremities). The research tool was the Tanita Viscan visceral and trunk fat analyzer AB140 using the abdominal bioelectrical impedance analysis (BIA) to estimate the visceral fat level (Vfat) and trunk fat percentage (Tfat). The AB140 analyzer also allowed the measurement of body composition of those individuals who could not assume an upright position. Our analyses revealed high and very high correlation coefficients between Vfat and WC (r=0.9), WHtR (r=0.7) and Tfat (r=0.9) whereas the correlation between Vfat and the BMI was weak, especially in the subgroup with Vfat < 13.5% (r=0.2). The subgroup with Vfat>13.5 exhibited a moderate-level relationship between the BMI and visceral fat increase. It was concluded that the BMI had a low sensitivity for predicting obesity risk in wheelchair rugby players after CSCI. The sensitivity of WC measurement was higher and thus, it may be stated that it constitutes an objective tool for predicting obesity risk in post-CSCI wheelchair rugby players.

## Introduction

Individuals suffering from dysfunctions and disorders of the locomotor system and those with cardiovascular diseases constitute, according to the Central Statistical Office, the largest disability groups in Poland. The predominant causes of motor organ disability are cervical spinal cord injuries (CSCI) sustained in various accidents and associated with lower extremity paresis and upper extremity paralysis. Progress in medicine and rehabilitation significantly increases chances of the victims of road traffic accidents and other tragic events to survive. The mortality of patients with CSCI depends on the level and time of injury (DeVivo et al., 1999; Garshick et al., 2005). Similar to or even more than able-bodied people, disabled individuals are also prone to civilization diseases – as a result of the injury itself as well as their post-injury lifestyle and behaviour patterns. The CSCI-related dysfunction frequently determines and generates other deficits and disorders. Post-traumatic complications affect almost all body systems and hence have the potential to worsen the subject’s condition and accelerate disease progression.

Compensatory processes may lead to the development of cardiovascular diseases - the leading cause of death among subjects who had sustained spinal cord injuries; the highest mortality rates have been reported following cervical spinal cord injuries (Frankel et al., 1998). The enforced sedentary lifestyle and muscle paresis below the level of injury are associated with adipose tissue accumulation in the trunk. Visceral fat accumulation, in turn, favors the development of cardiometabolic disease (Siemińska, 2007; Maruyama et al., 2008). Literature reports regarding this issue in post-CSCI populations are scarce. First of all, subjects after cervical spinal cord injuries suffer from a whole spectrum of pathological conditions and psychosocial complications, the severity of which undermines the importance of body weight and composition monitoring. Secondly, body weight measurement is difficult to carry out in this group. Thirdly, the estimation of the risk of obesity or cardiometabolic disease based on the commonly used indexes carries a high risk of being erroneous. Physicians and physical therapists are well aware of the problem; however, a number of doubts remain regarding possible solutions. Since the BMI tends to increase over time and after hospital rehabilitation, several researchers have emphasized the need for the BMI monitoring in subjects with spinal cord injuries (Mojtahedi et al., 2009). The value of anthropometric indicators of obesity in subjects with spinal cord injuries has also been called into question. Spinal cord injury-specific assessment tools should be sought, which, along with better understanding of the sequelae of excess body weight will lead to better targeting of prevention and treatment interventions (Rajan et al., 2008). Rajan et al. (2008) and Laughton et al. (2009) directly suggested that body mass index cutoffs should be lowered to better identify obese individuals with spinal cord injury. They believed that people with chronic spinal cord injury and BMI values > 22 kg m/2 should be considered as being at high risk of obesity and obesity-related chronic diseases. Thus, it seems well justified to undertake investigations aimed at the determination of the best and most effective tool to predict the risk of obesity and metabolic diseases in CSCI subjects.

We hypothesized that the Body Mass Index recommended by the WHO to diagnose obesity in general population had too low sensitivity in wheelchair rugby players after CSCI. The aim of the study was to verify the value of BMI and WC indexes as predictors of body composition in wheelchair rugby players who had sustained cervical spinal cord injury.

## Material and Methods

### Participants

The study group comprised 14 wheelchair rugby players, aged 32.6 ± 5.1 years, who had sustained CSCI and suffered from paralysis of lower limbs and paresis of upper extremities (tetraplegia). The level of lesion of wheelchair rugby players varied from C4 to C7. The most frequent cause of C4 or C5 spinal cord injury was head jump into water (63%), road traffic accident (32%), fall from height or other causes (5%). Minimum time after injury was 6 years. Mean training experience of the study participants was 7 ± 3.5 years (Table 1).

All subjects took part in wheelchair rugby training once a week for three hours. They had no history of cardiometabolic problems. Measurements were conducted by a qualified physiotherapist to minimize inter-observer differences.

Based on the International Wheelchair Rugby Federation classification system, the players were divided into two classes, ie., a low-point class with 0.5 to 1.5 points and a high-point class with 2.0 to 3.5 points. The wheelchair rugby athlete classification was based on a system developed by the International Stoke Mandeville Games Federation (ISMGF) and International Stoke Mandeville Wheelchair Sport Federation (ISMWSF) originally devised for tetraplegic players. High pointers had incomplete spinal cord injury; our study group comprised 21% of high point players (n=3). Other participants had sustained complete spinal cord injury.

All participants gave written consent to participate. The study was approved by the Ethics Committee and conformed to the standards set by the Declaration of Helsinki (Act No 9/2012 of 8 March 2012).

### Instruments, test procedures

Detailed data were obtained regarding general health condition, complaints and medications taken by the study participants. Subjects receiving long-term pharmacotherapy were excluded from our investigations. Body weight was measured using chair scales WE150P3 K (MENSOR, Poland). Considering the type and degree of disability as well as the likelihood of lower limb contractures or increased spasticity in individuals with cervical spine injury (Eriks-Hoogland et al., 2011), body height was measured in the supine position (to the nearest 1 mm). The final result was a mean of 3 measurements. WC was measured with an anthropometric non-stretch tape over light clothing. The Body Mass Index (BMI) was calculated, which is a useful measure of overweight and obesity (but cannot serve as an accurate indicator of body fat percentage) (Gupta at al., 2006). The BMI allows classification of able-bodied adults as underweight, normal weight, overweight or obese using the World Health Organization criteria. The World Health Organization regards a BMI greater or equal to 25 as overweight. An increased BMI constitutes a risk of cardiometabolic disease including obesity, type 2 diabetes mellitus and ischemic heart disease (Maruyama et al., 2008).

The research tool was the Tanita Viscan visceral and trunk fat analyser AB140 using the abdominal BIA (bioelectrical impedance analysis to estimate visceral fat level (Vfat) and trunk fat percentage (Tfat). The AB140 analyzer allowed the measurement of body composition and of those individuals who could not assume an upright position (Zamrazilová et al., 2010; Mateo-Gallego et al., 2012).

BIA is a reliable method for estimating body composition in post-SCI individuals; it is an alternative to dual-energy X-ray absorptiometry (DEXA) commonly used in clinical practice (Mojtahedi et al., 2009). A strong correlation was found between body composition assessed using DXA and BIA AB 140 measurements; BIA turned out to be more reliable compared to the skinfold thickness measurement (Mojtahedi et al., 2009). During the examination of subjects with increased spasticity, lower limbs were stabilized by the person performing the measurement. The exam lasted about 30 s, was non-invasive and in compliance with procedural requirements (after fasting) (Lewitt et al., 2007). Hip and waist circumferences (HC and WC) were taken in the lying position. Waist and hip circumferences were measured according to the recommended techniques, ie.: waist circumference – at the midpoint between the superior iliac crest and the lowest rib; hip circumference – around the greatest convexity of the gluteal muscles, below the iliac ala. In Europe, a WC of > 94 cm for men is considered a risk factor for obesity (Kuulasmaa, 2013). Based on the obtained results, the waist-to-hip ratio (WHR) was calculated which determines the obesity type (visceral, gynoid). The waist-to-height ratio (WHtR) was also determined; the ratio is presently deemed to be more effective in estimating obesity and the risk of cardiometabolic disease (Aschwell et al., 2012). Since the function of the respiratory muscles is frequently compromised in post-CSCI subjects (Scher, 1982), respiratory muscles efficiency (ie., a difference between chest circumference in full inhalation and exhalation – CCIE) was also analysed. The examinations were performed in the morning at the laboratory of the Academy of Physical Education in Katowice at the presence of a physician and a physiotherapist.

### Statistical analysis

Statistical analyses were performed with STATA statistical software (release 7; StataCorp, College Station, TX). All the data were tested for normal distribution. Mean values, standard deviations, maximal and minimal values were calculated for the analysed variables. 95% confidence intervals were used. The Student t test for unpaired data was used to evaluate differences in indexes and anthropometric characteristics for the BMI, WC, Vfat, Tfat between cut-off points for BMI>25kg/m^2^, WC>94, Tfat>26 and Vfat>13,5% (Tables 2 and 3). Significant bivariate correlations between the BMI, height, weight, hip circumference and % trunk fat were determined in our Vfat subgroups using the Pearson correlation coefficients (Table 4).

## Results

Obesity risk was determined using anthropometric indicators of obesity (BMI, WC, WHR, WHtR) according to reference range values recommended by the WHO (Table 2). Only 20% of wheelchair rugby players post-SCI were overweight. However, the difference between normal-weight and overweight participants was statistically significant (p<0.05). Mean values of WC significantly exceeded those of the WHO in 50% of our study subjects (p<0.05). Differences were also found regarding the visceral fat level and trunk fat percentage as determined with the Viscan AB140 analyzer. However, group size of individuals at increased risk of obesity depended on obesity prediction parameter used (Table 2).

Vfat of 13.5 was the visceral fat cut-off value based on measurements made with the Viscan AB140; significant differences were revealed regarding Tfat, BM and WC (p<0.01) between the Vfat<13.5 and Vfat>13.5 subgroups. Other predictors of obesity and cardiometabolic disease risk, ie., WC and WHtR, were also significantly different in the two subgroups (p<0.03). Similar observations were made with respect to WHR (p<0.04) and CCIE (p<0.03) whereas the BMI, HC and BH did not differ significantly between the study participants (Table 3).

Our analyses revealed high and very high correlation coefficients between Vfat and WC (r=0.9), WHtR (r=0.7) and Tfat (r=0.9), whereas the correlation between Vfat and the BMI was weak, and especially in the subgroup with Vfat < 13.5% (r=0.2). Hence, no relationship was observed between an increase in visceral fat and the BMI although the AB140 analyzer did indicate an excess of visceral fat. The subgroup with Vfat > 13.5 exhibited a moderate-level relationship between the BMI and visceral fat increase (Table 4).

## Discussion

Research has documented that overweight and obesity are commonly observed in the general population including individuals with disabilities related to musculoskeletal impairments. Muscle paresis below the injury site, a decrease in functional capacity and the enforced sedentary lifestyle cause that post-CSCI subjects are at high risk of obesity, and, consequently, cardiometabolic disease. It might seem that sport participation among wheelchair rugby players after CSCI should lower the risk compared to those who were physically inactive. However, a broad spectrum of post-injury complications frequently result in the development of chronic metabolic disease (Wilt et al., 2008; Kostovski, 2010). Spungen et al. (2003) observed that men with chronic spinal cord injury demonstrated 50% more adipose tissue for any given age compared with the able-bodied male reference population. It was also found that factors used to predict the risk of cardiometabolic disease increased during and after inpatient rehabilitation (de Groot et al., 2010).

Our own investigations clearly confirm the above mentioned findings. Although the BMI exceeded the reference range in only 20% of our study population, objective assessment of body composition using Viscan AB 140 revealed a significantly higher proportion of individuals at risk of visceral and trunk fat accumulation. An analysis of the sensitivity of several indicators of risk of obesity and cardiometabolic disease in wheelchair rugby players after CSCI demonstrated significant diagnostic accuracy of WC and WHtR. Our results are consistent with those of Buchholz and Bugaresti (2005), who found that the BMI was an insensitive marker of obesity in the SCI population. Hence, the conclusion of Laughton et al. (2009), who suggested lowering body mass index cutoff to <22 in this population, seems well-justified. The results of both research teams do indicate that WC is a more reliable obesity risk predictor in wheelchair rugby players post-SCI than the BMI. Our results confirm the findings mentioned above. Thus, although WC has not been formally approved as an alternative measure of visceral fat content, the preliminary results of this and other studies strongly suggest a relationship between WC and increased visceral and trunk adipose tissue accumulation in wheelchair rugby players who had sustained spinal cord injury. BMI sensitivity is too low for predicting obesity risk in post-CSCI wheelchair rugby players (BMI<25, r=0.2). Hence, the potential of WHtR, and the more so, since – according to the WHO – it further specifies cardiometabolic risk in the general population (Buchholz, and Bugaresti, 2005; Ashwell, 2009). Our own investigations clearly indicate significant correlations between WHtR and Vfat>13.5 (r=0.6) and Vfat<13.5 (r=0.4), ie., excess visceral fat is associated with a WHtR increase. WHtR of 55±5 observed in the Vfat<13.5 subgroup indicated overweight and was significantly different when compared to the value found in the Vfat>13.5 subgroup (p<0.03).

The respiratory muscle efficiency (CCIE) differed significantly in the Vfat>13.5 and Vfat<13.5 subgroups (p<0.03). Visceral fat affects the respiratory system mainly via an increase in intra-abdominal pressure and resultant impairment in the function of the diaphragm (Zieliński, 2012). Our investigations revealed negative correlation between visceral fat and respiratory muscles efficiency suggesting that increased accumulation of Vfat was associated with lower efficiency of the muscles. The severity of respiratory complications in post-CSCI wheelchair rugby players depends on the level of spinal injury (Tollefsen, 2012). Individuals with muscle paresis below the level of injury are at risk of fat tissue accumulation in the chest and abdomen area leading to abdominal obesity, which, in turn, increases the risk of cardiometabolic disease development.

## Conclusions

To our knowledge this is the first study with regard to the sensitivity of the BMI performed in wheelchair rugby players. Previous investigations concerning the usefulness of the BMI in post-CSCI individuals suggested that the major factor to decrease BMI sensitivity was the chronic enforced sedentary lifestyle. We would like to emphasize that the results of the present study clearly indicate that BMI sensitivity for predicting obesity risk is low in both sedentary and active subjects suffering from spinal cord injuries. Long-term and regular sports training does not seem to have a significant effect on visceral and trunk fat distribution in post CSCI athletes. Hence, our results are consistent with those of other authors who reported that the BMI had too low sensitivity for predicting obesity risk in the post-CSCI population, including wheelchair rugby players. We also confirmed that WC is a better obesity indicator in post-CSCI individuals irrespective of physical activity undertaken. Further investigations are needed to establish optimum indicators of fat tissue accumulation in the highly diversified wheelchair rugby players after CSCI. Spinal cord injury-specific assessment tools might facilitate assessment of the risk of cardiometabolic disease in wheelchair rugby players.

## Figures and Tables

**Table 1:** Characteristics of subjects after CSCI

**Variable**	**Min-max**	***X̄*^±S^**
Age (years)	25–40	32.6 ± 5.1
Time after injury (years)	6–24	12.5 ± 5.7
Age at the time of injury (years)	15–26	20.1 ± 3.6

**Table 2:** Obesity risk in the study group according to anthropometric indicators of obesity

Group size	BMI≤25	BMI>25	WC ≤94	WC >94	Tfat≤26	Tfat>26	Vfat≤13.5	Vfat>13.5

	n = 11	n = 3	n = 7	n = 7	n=5	n=9	n=8	n=6
*x̄* ± sd	21.8 ± 2.3	27.2 ± 0.8	85.7 ±4.1	98.7±5.2	25.1±3.1	37.5±4.6	10.4±1.8	17.5±4.7
T test	0.05^*^		0.01^*^		0.01^*^		0.01^*^	

Visceral fat level (Vfat) and trunk fat (Tfat) percentage (%)

* differences between the means were significant at p<0.01; p<0.05

**Table 3:** Verification of anthropometric indicators of obesity and cardiometabolic disease in Vfat subgroups(x̄ ± *sd*)

**Vfat**	**N**	**BM**	**BH**	**BMI**	**WC**	**HC**	**WHR**	**WHtR**	**CCIE**	**Kfat**
<13.5	8	68.88 ± 10.06	178.38 ± 5.40	21.60 ± 2.5	86.63±4.6	92.75±6.9	0.94±0.07	49±2	4.13 ± 1.9	25.10±3.3
T test p<0.05		0.01^*^	ns	ns	0.01^*^	ns	0.04^*^	0.03^*^	0.03^*^	0.01^*^
>13.5	6	82.67±4.5	183.3±10.1	24.78±3	99.67±5	98.33±7.5	1.02±0.06	55±5	2.00±1.3	35.47±6.5

*BM(kg)- body mass, BH(cm)-body height, BMI (kg/m)^−2^ body mass index, WC(cm)- waist circumference, HC(cm) - hip circumference, WHR (%)- waist-to-hip ratio, WHtR(%)- waist-to-height ratio,* CCIE (cm) - *difference between chest circumference in full inhalation and exhalation, Tfat(%)- trunk fat percentage, Vfat(%)- visceral FAT percentage;*

* differences between the means were significant at p<0.01; p<0.05; ns – non-significant

**Table 4:** Correlation coefficients in Vfat subgroups

Indicators	V fat <13.5	V fat >13.5	
	n=8	n=6	n=14
BM (kg)	0.5	0.2	0.7
BH (cm)	0.0	0.2	0.3
BMI (kg/m^2^)	0.2	0.6	0.6
WC (cm)	0.7	0.7	0.9
HC (cm)	0.2	0.3	0.4
WHR (%)	0.3	0.2	0.2
WHtR (%)	0.4	0.6	0.7
Tfat (%)	0.9	0.9	0.9
CCIE (cm)	−0.8	−0.3	−0.3

* significant p<0.05;

*BM(kg)- body mass, BH(cm)-body height, BMI (kg/m) ^−2^ body mass index,, WC(cm) - waist circumference, HC(cm) - hip circumference, WHR (%)- waist-to-hip ratio, WHtR(%)- waist-to-height ratio,* CCIE (cm) - *difference between chest circumference in full inhalation and exhalation, Tfat(%)- trunk fat percentage, Vfat(%)- visceral fat percentage;*
